# Ingestion of synthetic microparticles—microplastics and cellulose-based microfibers, by macroinvertebrates in the highly polluted Tietê River (São Paulo, Brazil)

**DOI:** 10.1007/s10661-026-15523-7

**Published:** 2026-06-01

**Authors:** Camila Magro, Marcos Gomes Nogueira

**Affiliations:** Department of Biodiversity and Biostatistics, Institute of Biosciences, State University of São Paulo, Botucatu, São Paulo 18618-689 Brazil

**Keywords:** *Corbicula fluminea*, Emerging contaminants, Ephemeroptera, Lotic ecosystems, Oligochaeta, µFTIR

## Abstract

Global contamination of aquatic ecosystems by synthetic microparticles has raised increasing concern, yet studies on their ingestion by freshwater invertebrates remain limited. Benthic macroinvertebrates, highly exposed to this kind of contamination, play a key role in aquatic food webs and nutrient cycling. This study investigated the ingestion of synthetic microparticles by benthic macroinvertebrates in the middle Tietê River basin, widely recognized as one of the most polluted rivers in Brazil. Samples were collected from the main river, a marginal lagoon, and a tributary under less human pressure. Contamination was expressive. A total of 129 synthetic microparticles were identified, from 0.02 to 1.78 particles per individual (mean of 0.5), between 7.14 and 900 particles per g of wet tissue. Size the mean values were 1.02 mm (main river), 0.81 mm (lagoon), and 0.64 mm (tributary). The presence of microplastics (polyester and polypropylene) was confirmed; however, cellulose-based fibers accounted for 90.7% of all identified synthetic microparticles. Ingestion rates did not differ significantly among environments, despite noticeable water-quality and hydrodynamics differences, suggesting that factors beyond local pollution levels may influence ingestion patterns. The filter-feeding bivalve *Corbicula fluminea* showed the highest ingestion values. Results provide the first evidence of synthetic microparticles ingestion by benthic macroinvertebrates in the Tietê River basin and establish an important baseline for future biomonitoring and risk assessment in Neotropical freshwater ecosystems.

## Introduction

Plastics are synthetic materials, mostly produced with petrochemicals polymers, widely used in the manufacture of innumerable products due to their favorable intrinsic properties, such as lightness, durability, and resistance (Gago et al., 2017; Geyer, 2020; Lusher et al., 2017; Rhodes, 2018). Due to these characteristics, the production of this material has increased over time, surpassing most other man-made materials (Geyer et al., 2017). However, the vast majority of plastics produced are not biodegradable, leading to their accumulation in landfills and all kind of environments for decades or even centuries (Geyer et al., 2017; MacLeod et al., 2021). The contamination of natural environments with plastic waste is an increasing concern and it is globally widespread (Suaria et al., 2020), even in remote areas such as deserts, mountain peaks, the deep ocean, and Arctic snow (MacLeod et al., 2021; Chauhan and Benerjee, 2025).

Microplastics (MPs) have been considered particles smaller than 5 mm that can originate from larger plastics through chemical, physical, oxidative, and ultraviolet degradation processes (Barnes et al., 2009; Bråte et al., 2017). They can also be introduced directly into aquatic environments as microfibers, microbeads, and pellets, which are components of personal care products (Andrady, 2011).

The MPs also coexist in the natural environments with other potentially adverse synthetic microparticles, particularly cellulose-based microfibers derived from processed textiles (Barrows et al., 2018; De Falco et al., 2019; Murphy et al., 2016). Although these fibers are not synthetic polymers, they are widely distributed due to human activities, especially domestic wastewater discharges (Di Lorenzo et al., 2023; Savoca et al., 2019). Given their similar morphology, these materials are often grouped together during visual analyses, even though they differ fundamentally in terms of chemical composition and environmental impacts (Remy et al., 2015). Therefore, distinguishing between microplastics and other synthetic microparticles is essential for accurately assessing contamination patterns and ecological risks (Athey & Erdle, 2022).

Currently, anthropogenic or synthetic particles, which include both plastic polymers and cellulose-based fibers, are ubiquitous in aquatic systems and represent a potential threat to biota (Masura et al., 2015; Gago et al., 2017; Athey & Erdle, 2022). Numerous studies have documented their ingestion by marine and freshwater organisms, including vertebrates and invertebrates (Caron et al., 2018; Cuthbert et al., 2019; Di Lorenzo et al., 2023; Moore et al., 2020; Neves et al., 2015; Silva et al., 2019; Silva-Cavalcanti et al., 2017; Vandermeersch et al., 2015). These particles can be transferred across trophic levels and may act as vectors for other contaminants, such as persistent organic pollutants, heavy metals, and antibiotics (Andrady, 2011; Barnes et al., 2009; Brennecke et al., 2016; Li et al., 2018; Lourenço et al., 2017; Moraes et al., 2024; Rochman et al., 2014). Despite increasing research efforts, most studies have focused on marine environments and temperate regions, leaving freshwater ecosystems understudied, particularly in the Neotropics (Fernandes et al., 2022; Grillo et al., 2022).

In South America, recent studies started to document the occurrence of microplastics and other synthetic particles in freshwater systems, including large river basins influenced by urbanization and industrialization (e.g., Mariano et al., 2025; Moraes et al., 2024; Nantege et al., 2023). Nevertheless, significant knowledge gaps persist, especially regarding the ingestion of these particles by benthic macroinvertebrates. This group plays a fundamental role in aquatic ecosystems, contributing to nutrient cycling and energy flow, and serving as a key link in food webs (Cummins, 1974; e.g. Rosenberg et al., 1994; Callisto et al., 2001; Carvalho & Uieda, 2004; Clarke et al., 2008). Macroinvertebrates are important indicators of environmental contamination due to their intrinsic association to the sediments, compartment where pollutants tend to accumulate (Mariano et al., 2025; Matsuguma et al., 2017).

The Tietê River, located in the most densely populated and industrialized region of Brazil, represents one of the most relevant cases of anthropogenic impact on freshwater ecosystems in the Neotropics (Moraes et al., 2024; Mariano et al., 2025; SOS MATA Atlântica 2025; 2026). Draining the São Paulo metropolitan region (22 million inhabitants), one of the largest urban and industrial centers in the world, the river receives substantial loads of untreated or partially treated sewage, industrial effluents, and urban runoff (Buckeridge & Ribeiro, 2018; Jacobi & Besen, 2011; Mariano et al., 2025; Rodgher et al., 2005; Silva et al., 2022; Tundisi et al., 2008). These conditions make the Tietê River a critical model for understanding the dynamics of synthetic microparticles contamination under intense environmental pressure, particularly in tropical regions where such studies remain scarce.

This study investigates the ingestion of synthetic microparticles, including microplastics and cellulose-based microfibers, by benthic macroinvertebrates in three contrasting environments within the middle Tietê River basin: the highly polluted main river channel, a marginal lagoon with limited hydrodynamics, and a tributary (Peixe River) with lower human influence. We hypothesized that ingestion rates would vary according to pollution levels, with higher contamination in organisms from more degraded environments. By integrating chemical identification (µFTIR) with ecological analysis, this study aims to contribute to a more comprehensive understanding of synthetic microparticles ingestion in Neotropical freshwater ecosystems.

## Material and methods

### Study area

The study was conducted in three freshwater environments located in the middle Tietê River basin, southeastern Brazil, representing a gradient of anthropogenic impact. The Tietê River is the main large river entirely within the State of São Paulo, 1.136 km long with an east-west orientation. It is located in the most populated and industrialized region of Brazil, making it the most emblematic Brazilian river in terms of environmental degradation (Buckeridge & Ribeiro, 2018; Jacobi & Besen, 2011).

The sampling area is located in the municipality of Anhembi, approximately 160 km downstream from São Paulo city, in a straight line, and 350 km when following the river’s meanders. Three distinct environments were selected for the study: the main course of the Tietê River (22°47′31.0″ S, 48°05′48.8″ W), a marginal lagoon (22°47′41.70″ S, 48°6′24.02″ W) (unnamed), with a small hydrodynamic connection with the river main channel (its only water source), and a tributary on its left bank, the Peixe River, less impacted by urban pollution (22°49′42.8″ S, 48°06′0.5″ W).

### Sampling procedures

**Fig. 1:**
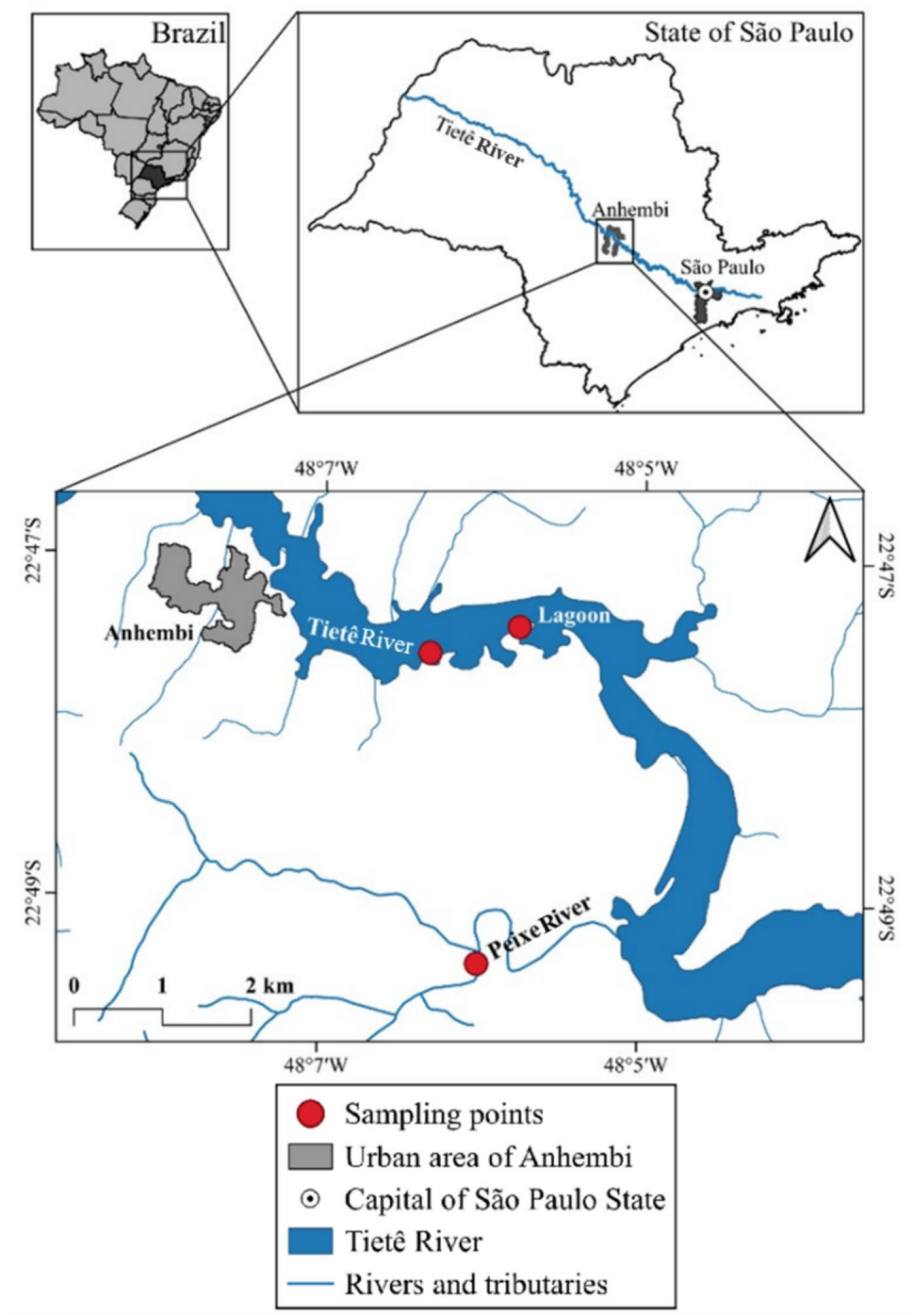
Map of the study area showing the three sampling locations: Tietê River, marginal lagoon, and Peixe River—in the middle Tietê River basin

**Fig. 2:**
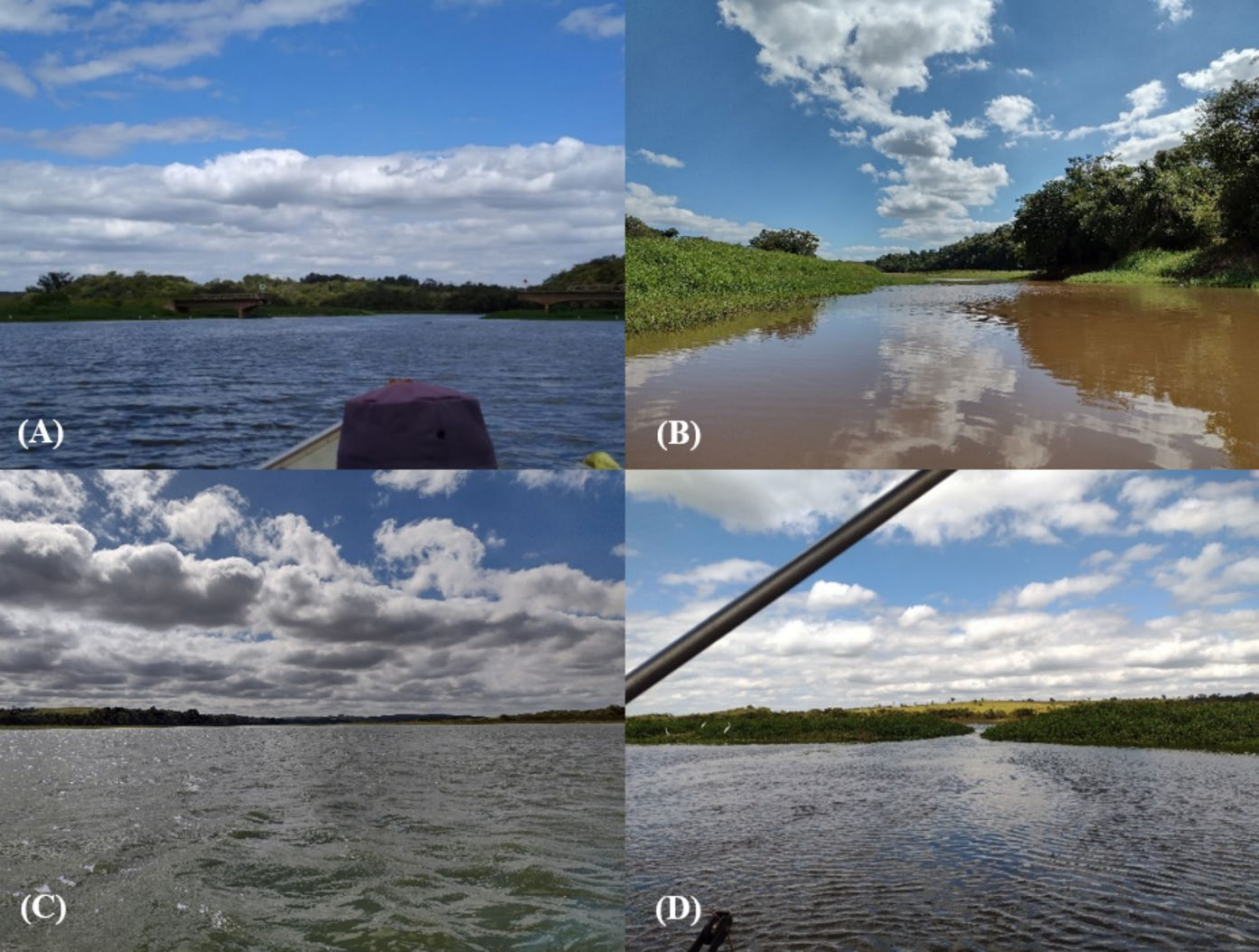
Sampling area: **A** Tietê River, **B** Peixe River, **C** marginal lagoon, and **D** marginal lagoon entrance

Sampling was conducted in the end of the rainy season (April 2021). The cumulative precipitation in São Paulo city (São Paulo-Mirante weather station; https://bdmep.inmet.gov.br/) for the previous three months (January, February, and March) was 534 mm. Longitudinal 1-km transects were established in three sites: Tietê River, marginal lagoon, and Peixe River (Figs. 1 and 2). Samples were taken in five equidistant points of each transect in a total of 15 points. Benthic macroinvertebrates were collected in triplicate at each point using a Van Veen dredge with an opening area of 0.0198 m^2^. The collected material was rinsed on-site using a 250-µm mesh net and immediately fixed with 8% formalin. In the laboratory, the samples were washed again with running water through a 250-µm mesh sieve and preserved in 70% alcohol.

### Ingestion analysis

Intact (non-fragmented) individuals from the dominant taxa of each site were selected for analysis: 50 Oligochaeta from Tietê, 50 Oligochaeta from lagoon, and in the case of Peixe River, which exhibits a higher macroinvertebrates diversity, 50 Mollusca bivalves of the species *Corbicula fluminea* (Müller, 1774), 50 Polymitarcyidae (Ephemeroptera), and 50 Oligochaeta.

Due to the small body size and low biomass of individual organisms, samples were processed using pooled individuals. For each taxon from each site, pools consisted of 50 individuals, resulting in a single composite sample per taxon-site combination. They were carefully rinsed with purified water to remove external particles. In the case of *C. fluminea*, shells were carefully removed, before weighing. Each pooled sample was weighed (wet weight) using an analytical balance (Denver Instrument P1-2250) and subjected to chemical digestion. Particle concentrations per individual were calculated as mean estimates by dividing the total number of particles detected in each pool by the number of individuals in the sample.

Samples (pooled individuals) were placed in small Petri dishes, one for each group of individuals, and exposed to a 15% KOH solution at 50 °C for 1 h for complete digestion (Dehaut et al., 2016). This protocol was adapted (reducing the time and temperature) to optimize processing time. However, always taking into account that complete digestion of the animal’s tissue had occurred (liquid homogeneous residuals), through cautious visual inspection. After digestion, the entire residue was carefully examined under a stereomicroscope to characterize, count, and measure the synthetic microparticles.

### Synthetic microparticles characterization

After digestion, the digested residuals were directly examined under a stereomicroscope (Zeiss SteREO Discovery.V20), without filtration. Particle identification was restricted to visually detectable items, following established morphological criteria (Hidalgo-Ruz et al., 2012; Norén, 2007). Only particles that could be clearly distinguished based on color, shape, and structural homogeneity were counted and measured. Very small particles (nanoparticles), below the visual detection limit, if present, were not computed. To ensure accurate material characterization, all recovered particles were analyzed using micro Fourier-Transformation Infrared (µFTIR) on a BRUKER Hyperion 2000 microscope to determine the chemical composition of the material (Prata et al., 2019). Spectra with the best statistical adjustments (Pearson correlation ≥ 0.7) were chemically identified using the database of the open-source program Open Specy (Cowger et al., 2021).

### Minimizing contamination

To minimize environmental contamination during the procedures described above, the use of plastic materials and instruments was strictly avoided. Work surfaces were regularly cleaned with 70% alcohol to reduce the potential for plastic contamination during the processes. Reagents were carefully prepared with ultrapure water and filtered to avoid introducing contaminants during experimental steps. Laboratory blanks were included and processed under the same conditions as the samples to monitor potential environmental contamination. During particle identification under the stereomicroscope, Petri dishes were covered with glass slides (Sarijan et al., 2018; Silva-Cavalcanti et al., 2017; Urbanski et al., 2020).

### Data analysis

The size of particles ingested by organisms was compared, considering the three locations, using the Kruskal-Wallis test, due to the non-normality of the data confirmed by the Shapiro-Wilk test.

Comparisons among taxa were performed, using the Kruskal-Wallis test, using particle concentration values (particles per individual and per g of wet tissue); however, these analyses are limited by the unequal distribution of taxa across sites (distinct assemblages structures). Only the taxon Oligochaeta was present in all environments, permitting only a descriptive comparison due to the absence of replicates.

To evaluate differences in the ingestion rates among environments, while minimizing taxonomic bias, only a descriptive comparison (replicates are missing—pooled samples) was performed for Oligochaeta, the only common taxon for the three sites.

Even considering the absence of real replicates per site and taxon, what presupposes caution about interpretation, correlation analyses between organism weight and particle concentration were performed using mean values derived from pooled samples. Data analysis was carried out in RStudio 4.3.3 (R Core Team, 2020).

## Results

**Fig. 3:**
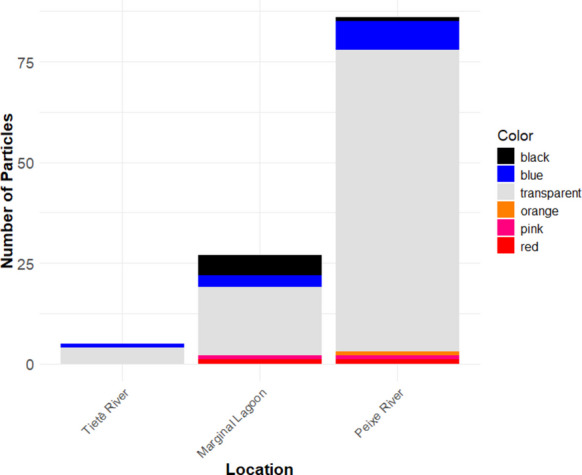
Distribution of colors of the macroinvertebrates ingested particles in the middle Tietê River basin

**Fig. 4:**
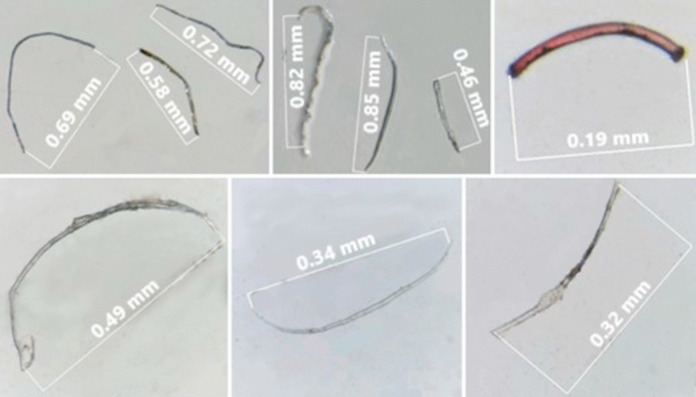
Selected images of the analyzed fibers from the middle Tietê River basin

**Fig. 5:**
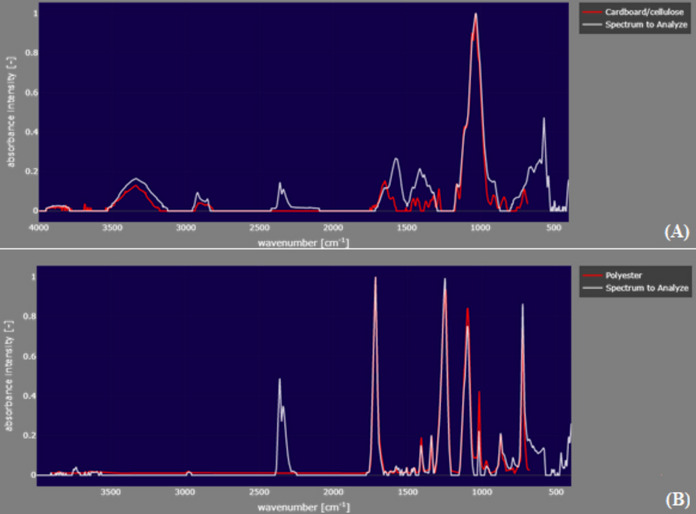
**A** Selected spectrum identified through µFTIR of a synthetic particle found in the bivalve *Corbicula fluminea* (*r* = 0.96). **B** Selected spectra identified through µFTIR of a synthetic particle found in the Oligochaeta (*r* = 0.96)

A total of 129 particles were identified, distributed among the colors transparent, blue, orange, black, red, and pink (Fig. 3). Fibers were the most abundant type, while only two fragments were recorded. Among the analyzed particles, 90.7% were composed of cellulose cardboard/cellulose while only three particles were confirmed as synthetic polymers, including polyester and polypropylene. Selected microscopic images and spectra of the analyzed particles are showed in Figs. 4 and 5. The considered cellulose fibers are likely of synthetic origin, as they were transparent or colored and did not break under needle stress.

Out of the total, 97 particles were found in organisms from the Peixe River (89 in *C. fluminea*, 1 in Polymitarcyidae, and 7 in Oligochaeta), 27 in Oligochaeta from the Lagoon, and 5 in Oligochaeta from the Tietê River. The characteristics and quantities of the ingested particles and respective locations are presented in Table 1.

**Table 1:** Characteristics of the material found in the macroinvertebrates from the middle Tietê River basin

Local	Taxon	Type	Color	Result
**Peixe River**	*C. fluminea*	Fiber	Transparent	Cellulose (67)
	Polymitarcyidae Oligochaeta	Fragment Fiber Fiber	Blue Orange Black Red Pink Blue Transparent Transparent	Cotton (8) Leaf (1) **Polyester** (1) **Polypropylene** (1) Cellulose (6) Cellulose (1) Cellulose (1) Cellulose (1) Cellulose (1) Cellulose (1) Cellulose (1) Cellulose (7)
**Tietê River**	Oligochaeta	Fiber	Transparent	Cellulose (4)
			Blue	Cellulose (1)
**Lagoon**	Oligochaeta	Fiber	Transparent	Cellulose (16)
		Fragment	Blue Black Pink Red Transparent	Cellulose (3) Cellulose (5) Cellulose (1) **Polyester** (1) Cellulose (1)

**Fig. 6:**
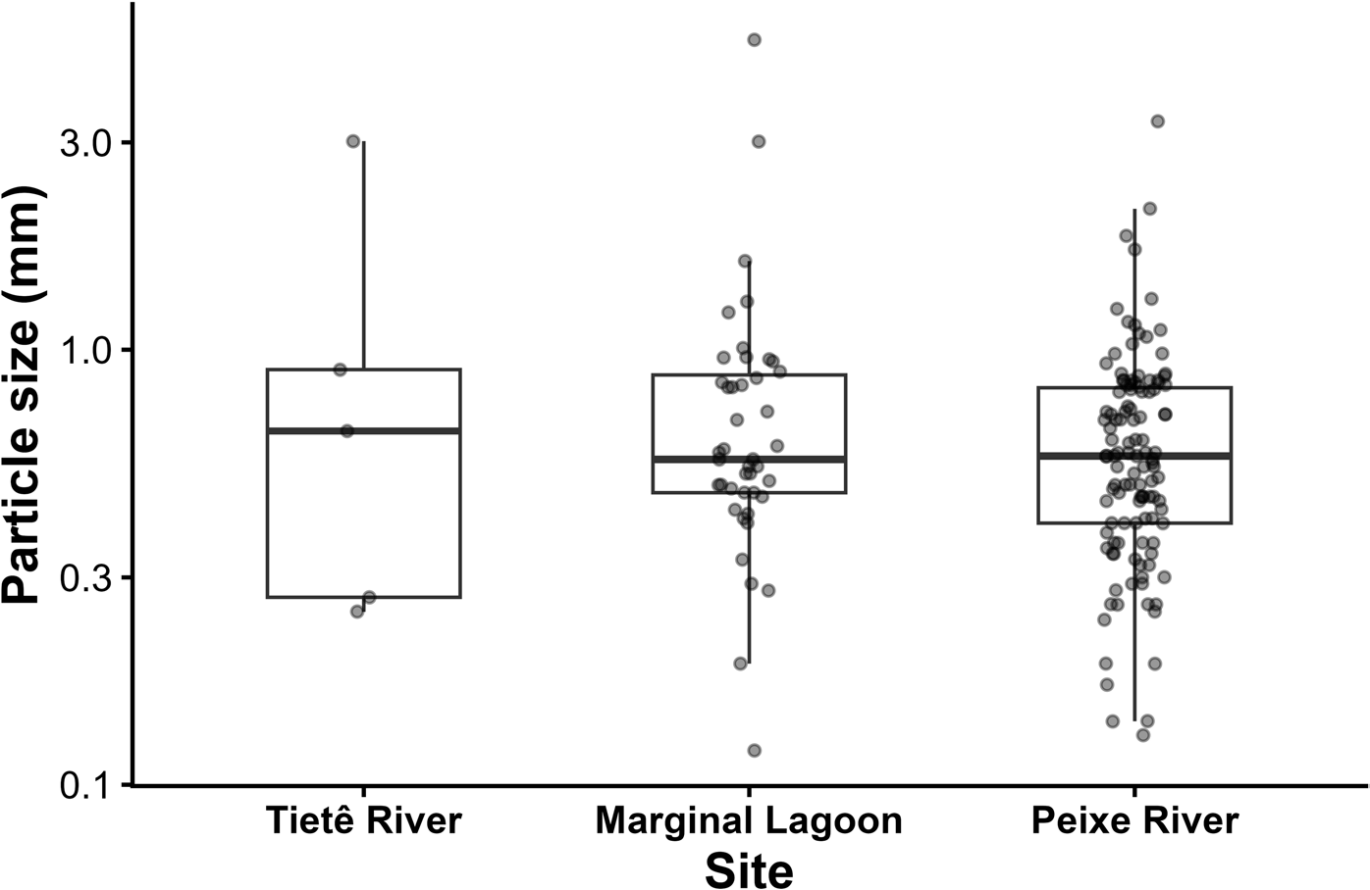
Distribution of particle size (mm) across sampling sites. Boxes represent median and interquartile range, and points represent individual particles. The *y*-axis is presented on a logarithmic scale

Particle sizes varied across samples, with the predominance of small particles and a limited number of larger ones (Fig. 6). The average particle size found was 1.02 ± 0.59 mm in the Tietê River, 0.81 ± 0.77 mm in the Lagoon, and 0.64 ± 0.39 mm in the Peixe River. No statistically significant differences in particle size were detected among sampling sites (Kruskal-Wallis test, *p* = 0.503). However, these results should be interpreted cautiously due to the limited replication associated with pooled samples.

Particle concentrations per individual and per g of wet tissue are presented in Table 2. Particle concentrations varied among sites, with higher values observed in the lagoon and Peixe River compared to the Tietê River. However, these differences are presented descriptively due to the lack of replication. No statistically significant differences in particle concentrations were detected among taxa (H = 4, *p* = 0.406). This analysis is constrained by the uneven distribution of taxa across environments, as only Oligochaeta were present in all three sites.

**Table 2:** Taxa, number of analyzed individuals, sample total weight and concentration of ingested particles from the middle Tietê River basin

Site	Taxon	No. analyzed individuals*	Total w.w. (g)**	Particle concentration/g w.w tissue	Particle concentration/individual
Peixe River	*C. fluminea*	50	2.67	33.3	1.78
	Polymitarcyidae	50	0.14	7.14	0.02
	Oligochaeta	50	0.07	100	0.14
Tietê River	Oligochaeta	50	0.08	62.5	0.1
Lagoon	Oligochaeta	50	0.03	900	0.54

*Represents the total number of individuals analyzed for each taxon. Note that individuals were grouped into pooled samples (up to 50 specimens each) for chemical digestion

**“Total sample weight” corresponds to the combined wet weight of all individuals included in each pooled sample prior to chemical digestion

Considering the taxon that is common to the three sites, Oligochaeta, differences in particle concentrations among sites were observed. Much higher concentrations were recorded in the lagoon, followed by Peixe River, while lower values were observed in the Tietê River. However, these differences cannot be statistically tested, due to the lack of replication associated with pooled samples.

Additionally, no correlation was found between the average weight of the taxa and the average particle concentration in their tissues (*ρ*[33] = −0.9, *p* = 0.083). Given the small sample size and the use of pooled data, this result should be interpreted with caution.

## Discussion

The ingestion of synthetic microparticles by benthic macroinvertebrates was observed across all taxa and environments in the middle Tietê River basin. Despite the contrasting environmental conditions (water-quality, hydrodynamics) among the three studied sites (Mariano et al., 2025), particle ingestion did not differ significantly between the highly polluted Tietê River, its adjacent lagoon and the low-impacted tributary. Similar result was reported by Cesarini et al. (2025), that found no significant differences in the ingestion rates of estuarine bivalves exposed to different levels of human pressure. These results suggest that additional factors, besides the local pollution, might play an important role in determining ingestion patterns.

Previous work conducted in the same study area showed marked differences in sediment contamination. In that study, the highest concentrations of synthetic particles were recorded in the Tietê River (~190,000 items kg⁻^1^), followed by the marginal lagoon (~180,000 items kg⁻^1^), and substantially lower values in the Peixe River (~10,000 items kg⁻^1^) (Mariano et al., 2025). Based on these findings and on the expected positive relationship between environmental contamination and ingestion (Nantege et al., 2023), a higher ingestion rate was anticipated in the Tietê River. This expectation is also supported by studies on fish from the same basin, where higher particle loads were reported in individuals from the Tietê River compared to the Peixe River (Urbanski et al., 2020). However, the absence of a clear pattern for macroinvertebrates indicates that factors such as feeding strategy, particle selectivity, and environmental availability may modulate the synthetic microparticles ingestion dynamics.

A key finding of the present study is the considerable higher predominance of cellulose-based fibers (90.7%) among the identified particles, while only three particles were confirmed as synthetic polymers (polyester and polypropylene). This result indicates that anthropogenic contamination in the studied environments is largely driven by non-plastic materials rather than conventional microplastics. These cellulose-based fibers share physical similarities with microplastics, making visual inspection insufficient for distinguishing between the two materials (Remy et al., 2015). Thus, the use of advanced methods, such as µFTIR, was crucial for confirming the chemical composition of the particles, as strongly recommended by Prata et al. (2019).

In the context of the Tietê River basin, several potential sources may explain the high occurrence of cellulose fibers. The river receives substantial inputs of untreated or partially treated domestic sewage, which is known to contain large quantities of cellulose-derived materials such as toilet paper and other sanitary products (Murphy et al., 2016; Quevedo and Silva Paganini, 2017; De Falco et al., 2019). In addition, textile effluents from urban and industrial areas may contribute both natural and regenerated cellulose fibers, which are widely used in fabric production (Barrows et al., 2018; Di Lorenzo et al., 2023; Liu et al., 2023; Savoca et al., 2019). These combined sources are consistent with the high demographic and industrial pressure in the region and reinforce the role of wastewater and diffuse pollution as major pathways of synthetic particles. Even in less impacted environments, such as the Peixe River, previous studies have reported unexpectedly high fiber concentrations, potentially associated with fishing activities (e.g., gillnet losses) and diffuse inputs from rural communities (Mariano et al., 2025).

The occurrence of synthetic microparticles in environments with lower levels of urbanization, such as the Peixe River, may also be explained by processes widely reported in the literature. River systems act as transport pathways, allowing particles to disperse over long distances from their original sources (Mariano et al., 2025; Moraes et al., 2024). In addition, atmospheric transport and deposition may contribute to the input of fibers even in remote or less impacted areas (Hee et al., 2023; Napper et al., 2023). Furthermore, environments with reduced hydrodynamics, such as marginal lagoons, may function as depositional zones where particles accumulate over time (Edo et al., 2020; Matsuguma et al., 2017). Diffuse sources, including rural settlements, fishing activities, and inadequate waste disposal, may further contribute to contamination in these areas. These processes help explain why particle occurrence does not always directly reflect proximity to major urban centers.

Particle size distribution showed a predominance of smaller particles, for the three analyzed environments. This result is consistent with previous studies in freshwater systems, where continuous inputs and fragmentation processes lead to the accumulation of small particles (Horton & Dixon, 2018). Similar size distributions have been reported for sediments and water in the Tietê River basin (Mariano et al., 2025).

In terms of color, transparent fibers were the most frequent, followed by blue particles, which is also consistent with previous findings in the region (Mariano et al., 2025), as well as with other studies involving freshwater invertebrates (Awuor et al., 2020; Bertoli et al., 2022; Hurley et al., 2017).

No significant differences in particle size were detected among taxa, and no correlation was observed between organism weight and particle ingestion. Similar finding was seen by Bertoli et al. (2022), when compared distinct macroinvertebrates trophic guilds from an Italian river. Nevertheless, contrasting results have been reported elsewhere (Di Lorenzo et al., 2023). It is important to note that our analyses are limited by the use of pooled samples, which reduces statistical power and prevents the assessment of individual variability. Therefore, the absence of significant patterns should be interpreted cautiously.

Oligochaeta was the only dominant taxon present in all three analyzed environments. Despite the impossibility to test statistically the differences, much higher ingestion rate of synthetic microparticles for this taxon was observed in the lagoon, 14.4 times higher (mean values of microparticles/individual) compared to adjacent Tietê River. Visually, the organisms appeared to differ in size among the three locations; however, not all individuals were measured, so it is not possible to assert a correlation between organism size and particle ingestion. Smaller individuals were observed in the Peixe River, while those from the Lagoon and Tietê River appeared to be larger. Initially, it was assumed that organism size would influence the size of ingested particles, as reported by Scherer et al. (2017) and Nantege et al. (2023). Factors, such as environmental particle availability, hydrodynamics (lotic versus lentic conditions), and individuals developmental stage, may play a more relevant role in determining ingestion patterns.

The filter-feeding bivalve *Corbicula fluminea* exhibited the highest concentration of ingested particles (1.78 particles/individual), with a similar value to the one found by Cesarini et al (2025) (1.96 particles/individual) for the bivalve *Scrobicularia plana* in a Spanish coastal river. High contamination in bivalves aligns with previous studies and evidences the vulnerability of filter feeders to this type of pollution (Awuor et al., 2020; Cesarini et al., 2025), in addition to the detritivores (Di Lorenzo et al., 2023). This group is particularly vulnerable due to its feeding strategy, which involves the continuous filtration of suspended particles, increasing the likelihood of ingestion of synthetic materials.

## Conclusion

This study provides the first evidence of synthetic microparticles (microplastics and cellulose-based microfibers) ingestion by benthic macroinvertebrates in the highly polluted Tietê River basin, one of the most impacted freshwater systems in Brazil. The predominance of cellulose-based fibers demonstrates that contamination is not restricted to conventional microplastics, but also involves a substantial contribution of non-plastic solid waste materials. The widespread ingestion observed highlights the vulnerability of aquatic biota and establishes an important baseline for future biomonitoring and risk assessment in Neotropical freshwater ecosystems. These findings emphasize the need for improved wastewater treatment, better control of industrial and textile effluents, and the implementation of monitoring strategies that include both plastic and non-plastic contaminants. Results reinforce the need to expand the current microdebris framework to better evaluate the complexity of contamination in freshwater environments.

## Data Availability

No datasets were generated or analysed during the current study.

## Declarations

All authors have read, understood, and have complied as applicable with the statement on “Ethical responsibilities of Authors” as found in the Instructions for Authors.
